# Case Report: Spinal cord stimulation for neuropathic pain from spinal cysticercosis

**DOI:** 10.3389/fnins.2026.1742006

**Published:** 2026-02-24

**Authors:** Mali Guo, Yixin Zhang, Meirui Li, Guangjian Zhang

**Affiliations:** 1 Yanbian University, Yanji, Jilin, China; 2 Department of Pain, Yanbian University Hospital, Yanji, Jilin, China

**Keywords:** case report, neuropathic pain, refractory pain, spinal cord cysticercosis, spinal cord stimulation

## Abstract

**Rationale:**

Spinal cord cysticercosis, an exceptionally rare form of neurocysticercosis (NCC), often leads to refractory neuropathic pain and neurological deficits. Current treatments (e.g., antiparasitics or decompressive surgery) may fail to alleviate symptoms, necessitating alternative strategies. Spinal cord stimulation (SCS) is well-established for chronic pain but has rarely been reported for NCC-related pain.

**Patient concerns:**

A 69-year-old female presented with an 8-year history of progressively worsening trunk and limb pain (burning sensation, VAS 8/10), sensory abnormalities, and lower limb weakness (MMT 3/5). She had undergone laminectomy for spinal cysticercosis in South Korea, but pain persisted post-operatively.

**Diagnoses:**

MRI revealed residual cystic lesions with arachnoiditis at T12-L2, consistent with inactive spinal cysticercosis. Electromyography confirmed mixed sensorimotor polyneuropathy.

**Interventions:**

After multidisciplinary evaluation, a percutaneous SCS electrode was implanted at T10. Intraoperative testing achieved 80% pain coverage. Parameters were titrated post-operatively (frequency: 40 Hz, pulse width: 300 μs).

**Outcomes:**

At the 1-week follow-up, the patient reported significant pain relief, with her visual analog scale score dropping to 2 out of 10. Motor strength improved to grade 4 out of 5 on manual muscle testing, and sensory function returned to normal. These benefits persisted at the 3-month follow-up, accompanied by a 75% reduction in opioid requirements, measured in morphine milligram equivalents.

**Lessons:**

SCS may be a viable option for spinal cysticercosis-induced central neuropathic pain when conventional therapies fail. Its dual benefits (pain relief + functional recovery) warrant further study in NCC-related complications. Early SCS intervention could prevent chronic disability in similar cases.

## Background

Spinal cord cysticercosis, a rare form of neurocysticercosis (NCC) caused by Taenia solium larvae, accounts for only 1.5%-3% of NCC cases, with most spinal NCC occurring in the subarachnoid space (Alsina et al., 2002). Patients with spinal NCC often develop functional impairments due to spinal cord or nerve root damage, presenting with symptoms such as pain, muscle weakness, motor deficits, and sensory disturbances (Colli et al., 2002). There is no consensus on the optimal treatment, with both pharmacological and surgical options available. Studies have shown that patients undergoing laminectomy often experience no significant neurological improvement or even deterioration post-operatively (Colli et al., 2002). Neuropathic pain, caused by damage or dysfunction of the central or peripheral nervous system (Baron et al., 2010), can be effectively managed with spinal cord stimulation (SCS), a proven and safe therapy for refractory chronic pain in the trunk and limbs, including post-surgical and neuropathic pain (Rock et al., 2019). Here, we report a rare case of central neuropathic pain caused by spinal cord cysticercosis successfully treated with SCS, with significant symptom improvement. To our knowledge, this is the first documented case of SCS application in such a patient.

## Case report

**Figure 1:**
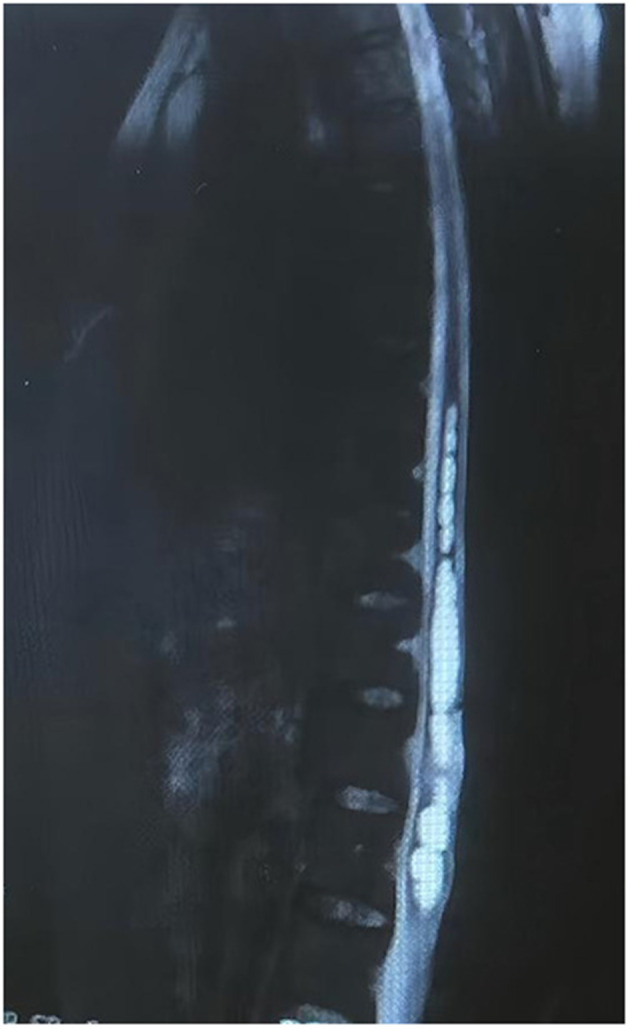
Spinal MRI provided by the patient, revealing an intradural cysticercal lesion.

A 69-year-old female patient was admitted to our Pain Management Department on September 6, 2024, with a chief complaint of “trunk and limb pain for 8 years.” Eight years prior, she developed generalized, migratory pain without an obvious cause. The pain was described as pulling, burning, and paroxysmal electric shock-like sensations, distributed in patches across the trunk and limbs. Subsequently, the patient was referred to and evaluated at a tertiary hospital in Korea, including spinal MRI (Figure 1), led to the diagnosis of spinal cord cysticercosis. The patient underwent laminectomy, which was uneventful, with good post-operative recovery. However, the pain persisted and progressively worsened in both intensity and extent, eventually involving the entire trunk and limbs.

The pain was accompanied by sensory disturbances, progressive lower limb weakness, distal numbness, and coldness, predominantly on the right side. Symptoms in the face and left upper limb were milder. The pain was spontaneous, without a clear pattern or identifiable aggravating or alleviating factors.

Clinical examination revealed hyperalgesia in the right thigh, left lower limb, and trunk; sensory deficits in the right calf and foot; and quadriceps muscle strength graded III on the right and IV on the left. Muscle tone and physiological reflexes were normal, and pathological signs were absent. The VAS (Visual Analog Scale) score ranged from 6 to 7.

**Figure 2:**
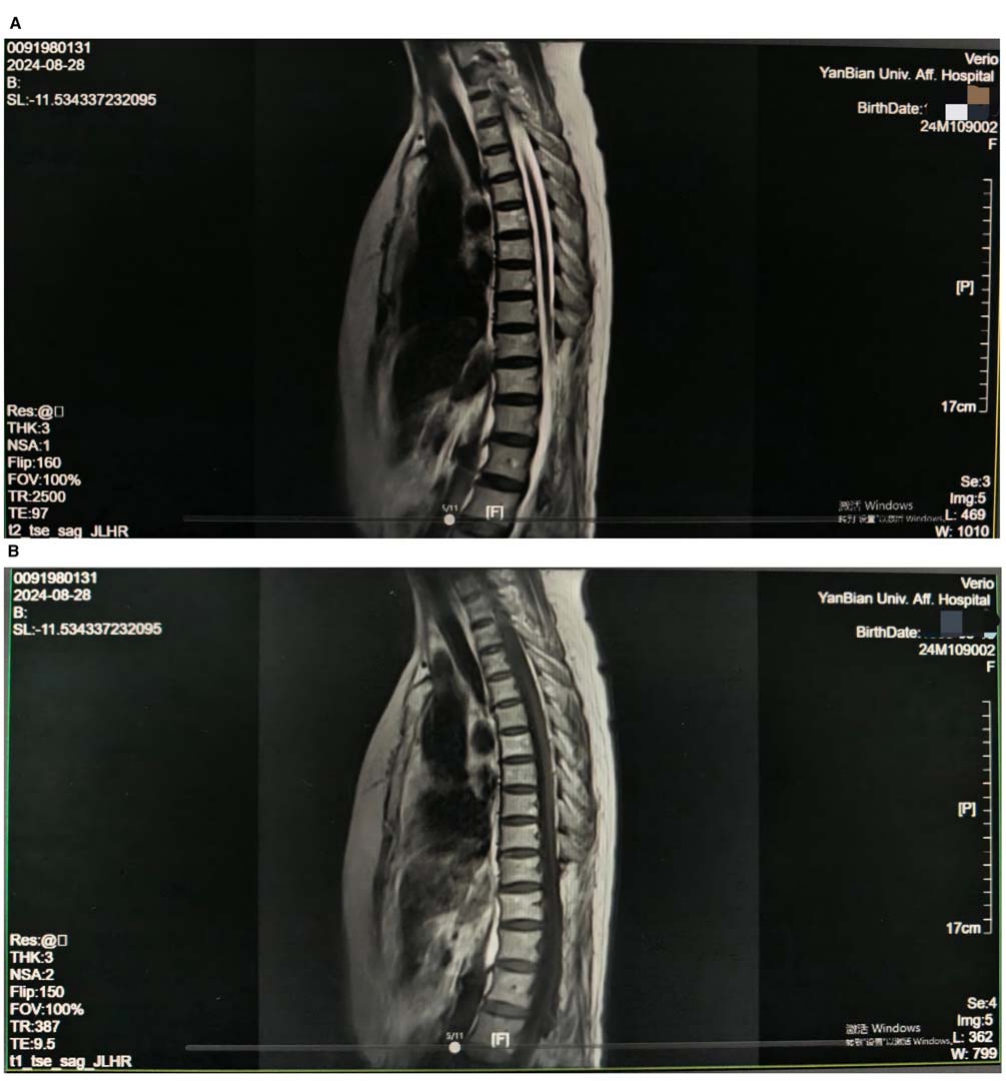
**(A, B)** Pre-operative spinal MRI from this admission, showing post-operative changes in the thoracolumbar region with absent spinous processes and laminae at T10, T11, T12, L1, and L2, along with heterogeneous signals.

Spinal MRI (August 28, 2024, at our hospital) showed post-operative changes in the thoracolumbar region, with normal thoracic curvature. The spinous processes and laminae of T10, T11, T12, L1, and L2 were absent, with heterogeneous signals and strip-like cerebrospinal fluid signals in the posterior cauda equina, consistent with post-operative changes. No obvious destruction of vertebral bodies, disc herniation, or intramedullary spinal cord abnormalities were observed (Figure 2). Other pathological causes of pain were excluded based on these findings.

The patient was diagnosed with 1. Central neuropathic pain and 2. Post-laminectomy syndrome. She had undergone various pain treatments, including physical therapy, interventional pain management, and multiple analgesic regimens, all of which failed to achieve significant pain relief.

Based on the patient's current symptoms and the absence of significant surgical contraindications following comprehensive evaluation, temporary dual-lead spinal cord stimulation implantation was considered to alleviate pain and improve sensory modulation in the limbs. Given the MRI findings indicating laminectomy from the L2 vertebra up to the T10 segment, the implantation site was determined to be at the T10 level.

**Figure 3:**
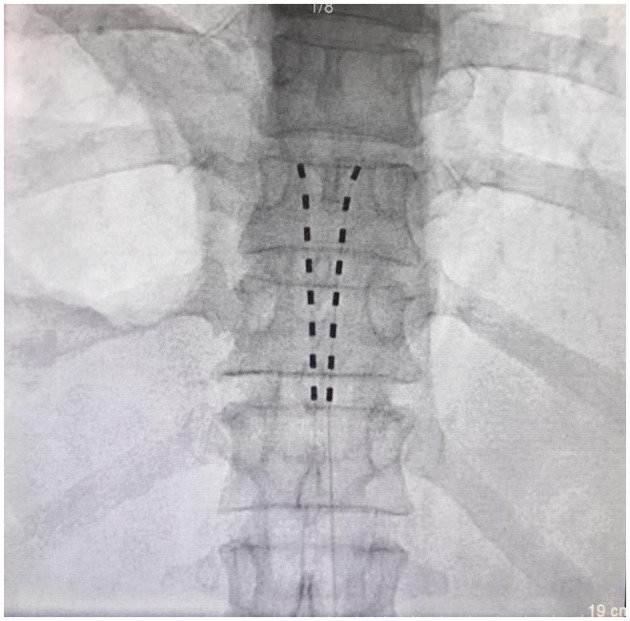
Fluoroscopic image showing the tips of the percutaneous spinal stimulation electrode leads positioned at the inferior margin of the L2 vertebral body on both sides of the spinous process.

On September 10, 2024, from 14:15 to 14:55, the patient underwent temporary dual-lead spinal cord stimulation implantation under local anesthesia, conscious monitoring, and non-invasive observation (Figure 3). Guided by DSA, the L5/S1 interlaminar space was identified, with the left interlaminar space selected as the puncture site. Following local anesthesia, a puncture needle was inserted under DSA guidance, and the needle tip was confirmed to be in the epidural space. After removing the needle core, aspiration showed no blood or cerebrospinal fluid, and 1 mL of iohexol (contrast agent) was injected, with DSA confirming epidural distribution.

An electrode lead was carefully advanced under DSA guidance, with its direction adjusted to align parallel to the spinous process on the right side, stopping at the lower edge of the right T10 vertebral body. The lead was connected to a multi-electrode test cable and a testing stimulator. At stimulation parameters (1.25 V, 200 μs, 50 Hz), the patient reported tolerable paresthesia (aching or tingling) in the original pain region. After securing the electrode lead with sutures and applying sterile dressings, the puncture site was compressed for 5 min. The procedure was then repeated for the second electrode, positioning its tip at the lower edge of the left T10 vertebral body.

## Outcomes

Following treatment, by September 11, 2024, the patient reported an 80% reduction in trunk and limb pain. Physical examination revealed decreased pain sensitivity in the right thigh, left lower limb, and trunk. Quadriceps muscle strength was graded as III on the right and IV on the left, with normal muscle tone, physiological reflexes, and no pathological signs. The VAS score improved to 0–2.

The patient was discharged on the second post-operative day. During the hospital stay, stimulation parameters were adjusted based on patient feedback, with discharge settings at 1.20 V, 200 μs, and 50 Hz. At a follow-up visit 1 week post-surgery, the patient reported complete resolution of trunk and limb pain, with a 100% reduction in symptoms and a VAS score improvement from 7/10 to 0/10. Muscle strength, limb sensation, numbness, and coldness in the lower extremities showed significant improvement, allowing for removal of the stimulator during the outpatient visit (Table 1).

**Table 1:** Time point and condition.

**Time point**	**Post-surgery (8 years ago)**	**2 Before treatment in our hospital**	**After stimulator implantation**	**7 days after implantation (follow-up and removal)**
Condition	Pain range initially decreased but gradually increased in severity and range.	Decreased sensation in lower legs and feet, numbness and coldness in distal lower limbs, hyperalgesia and spontaneous pain, VAS score 6–7.	Pain relief, VAS score 1–2.	Significant improvement in limb muscle strength, sensation, numbness, and coldness; VAS score 0–1.

## Discussion

We present a unique case of central neuropathic pain caused by spinal cord cysticercosis successfully treated with spinal cord stimulation (SCS). 1 Spinal cord cysticercosis often results in spinal cord and nerve root damage, 2 potentially due to mass effect with spinal cord compression, cerebrospinal fluid obstruction, or immune-mediated arachnoiditis and basal meningitis. Surgical intervention is typically recommended (Rajshekhar, 2010). Neurological deficits caused by acute mass effect may improve after decompression, whereas chronic dysfunction, such as myelitis, is often associated with poor prognosis (Alsina et al., 2002). Furthermore, central nervous system sensitization may persist even after removal of the underlying cause (Baron et al., 2010). This aligns with our patient's presentation, consistent with chronic central neuropathic pain.

Spinal cord stimulation (SCS), a neuromodulation technique, is rooted in Melzack and Wall's “Gate Control Theory of Pain,” which has evolved significantly over time. Its efficacy was first demonstrated in the 1970s in a case of chronic cancer pain successfully treated with SCS, providing early clinical support. Beyond the gate control mechanism, SCS exerts its pain-modulating effects through multiple pathways, including regulation of the descending inhibitory system, noradrenergic and serotonergic pathways, and improving blood flow. Additionally, SCS influences neuronal excitability and conduction by modulating electrophysiological activity, alleviating sensory abnormalities and numbness. Numerous studies have confirmed its effectiveness in treating chronic refractory pain in the trunk, limbs, and neuropathic conditions (Rock et al., 2019; Ali and Schwalb, 2024), consistent with our findings.

Moreover, research suggests that SCS may activate residual sensory fibers in the dorsal spinal root, stimulate spinal reflexes, and engage neuronal networks in surrounding tissues, thereby aiding in the contraction and relaxation of paralyzed muscles. This indicates a potential role for SCS in restoring motor control in patients with spinal cord injuries. However, the optimal system selection, waveform, and parameter settings remain under investigation (Minassian et al., 2016). In this case, follow-up revealed notable improvements in the patient's lower limb strength and motor flexibility.

In conclusion, the therapeutic outcome in this case demonstrates the efficacy of SCS in treating neuropathic pain caused by spinal cord cysticercosis. The benefits extend beyond pain relief to include recovery of impaired motor function, providing a valuable reference for managing refractory pain in similar patients.

## Conclusion

This case highlights the successful application of spinal cord stimulation in managing refractory neuropathic pain secondary to spinal cord cysticercosis—a rare and challenging condition with limited treatment options. Beyond significant pain relief, SCS contributed to the recovery of motor function and sensory deficits, suggesting its potential role in modulating both nociceptive and motor pathways in chronic spinal cord disorders.

The observed improvements align with established neuromodulatory mechanisms of SCS, including gate control theory, descending inhibition, and enhanced spinal blood flow. Importantly, this case underscores that SCS may offer dual benefits (pain control and functional restoration) even in late-stage disease, where conventional therapies often fail. Future studies should explore optimal SCS parameters and timing of intervention in neurocysticercosis-related neurological deficits.

Our findings support SCS as a viable therapeutic option for similar cases, particularly in endemic regions where spinal cysticercosis remains an underrecognized cause of disability. Early referral for neuromodulation evaluation could improve outcomes in patients with progressive or treatment-resistant symptoms.

## Data Availability

The original contributions presented in the study are included in the article/supplementary material, further inquiries can be directed to the corresponding author.
